# Fluid dynamics during bleb formation in migrating cells *in vivo*

**DOI:** 10.1371/journal.pone.0212699

**Published:** 2019-02-26

**Authors:** Mohammad Goudarzi, Aleix Boquet-Pujadas, Jean-Christophe Olivo-Marin, Erez Raz

**Affiliations:** 1 Institute of Cell Biology, ZMBE, Münster, Germany; 2 Institut Pasteur, Bioimage Analysis Unit, Paris, France; 3 CNRS UMR3691, Paris, France; University of Bonn, GERMANY

## Abstract

Blebs are cellular protrusions observed in migrating cells and in cells undergoing spreading, cytokinesis, and apoptosis. Here we investigate the flow of cytoplasm during bleb formation and the concurrent changes in cell volume using zebrafish primordial germ cells (PGCs) as an *in vivo* model. We show that bleb inflation occurs concomitantly with cytoplasmic inflow into it and that during this process the total cell volume does not change. We thus show that bleb formation in primordial germ cells results primarily from redistribution of material within the cell rather than being driven by flow of water from an external source.

## Introduction

Cell migration is instrumental during normal development and for homeostasis in the adult organism [1–4]. When misregulated, cell migration is associated with pathological conditions such as inflammation and cancer cell metastasis [5, 6]. Understanding the cellular events contributing to the migration of cells is thus of general interest in biology and medicine. At the biophysical level, the precise mechanisms contributing to translocation of the cell body, a process accompanied by shape changes and flow of material, are not fully understood. Cells employ two main migration strategies, with certain cell types capable of alternating between two migration modes [7–9]. In the first mode of migration, cells make use of actin polymerization at the cell front as a means for pushing the membrane forward [10]. The other migration strategy, used by different cell types including zebrafish primordial germ cells (PGCs) involves the formation of blebs as a mean for translocation of the cell body [11–15].

Blebs are spherical protrusions demarcated by the plasma membrane that detaches from the underlying acto-myosin cortex [13, 16]. A characteristic feature of blebs is the rapid change in cell shape at the site where the protrusion occurs and what appears to be an inflation of part of the cell. Understanding the mechanisms contributing to the formation of the bleb requires the identification of the source of membrane that envelops it and the source of the material driving protrusion expansion. While we have recently shown that a local release of membrane folds around the site of bleb formation accounts for the apparent increase in cell surface [17], the origin of the material that fills in the bleb is still controversial.

According to previous experimental and theoretical work, blebbing is not correlated with significant alterations in cell volume [11, 18, 19]. However, the measurements in those studies were conducted on cell fragments exhibiting extensive non-directional blebbing *in vitro*, or, when studies were conducted in an *in vivo* setting, the frequency of image capture was low and not correlated directly to the precise time of formation of specific blebs [11, 18, 19]. This uncertainty motivated a recent study performed in the *in vivo* context of germ cell migration within the developing zebrafish embryo, which challenges the notion of a constant cell volume during blebbing [20]. In this study, the formation of blebs was reported to be correlated with a significant increase in cell volume, with water influx into the cell proposed to account for the elevation in overall cell volume. According to this proposition, the influx of water into the cells requires channels called aquaporins (Aqp), specifically the isoforms Aqp1 and Aqp3. An untested prediction of the current model is that the formation of the bleb is associated with a pattern of water flow from out of the cell inwards, with bleb inflation representing a rather local event.

To critically examine the opposing views concerning the topic of fluid flow patterns and volume changes upon bleb formation, we employed blebbing zebrafish germ cells as an *in vivo* model for this process. We conducted detailed, high temporal resolution volume measurements, determined the pattern of cytoplasm flow within cells during bleb inflation and evaluated the possible role of aquaporins in the process.

## Methods

### Zebrafish strains

Zebrafish (*Danio rerio*) embryos of transgenic fish carrying Tg(*kop-mcherry-f-3'nanos3UTR*) that leads to mCherry expression on the plasma membrane of PGCs [21] were used as wild-type fish. Fertilized eggs were collected and raised at 25°C, 28°C or 32°C (different temperatures were used to slow or accelerate the embryonic development), with the measurements themselves conducted at 25°C. Embryos were kept in 0.3x Danieau’s solution [17.4mM NaCl, 0.21mM KCl, 0.12mM MgSO_4_·7H_2_O, 0.18mM Ca(NO_3_)_2_, 1.5mM HEPES (pH 7.6)]. The general fish maintenance at the Institute follows the regulations of the LANUV NRW and is supervised by the veterinarian office of the city of Muenster.

### Spinning disk microscopy

Embryos were imaged using a Carl Zeiss Axio imager Z1 microscope equipped with a Yokogawa CSU X.1 spinning-disk unit. Sample temperature was kept constant using a heated stage (PECON, TempController 2000–2). Imaging was performed using a 63x NA = 1.0 water immersion objective, a Hamamatsu Orca flash 4.0 camera and the Visitron Systems acquisition software (Visi-View).

### Volume measurements

PGCs of 12 hours post fertilization (hpf) old embryos from the Tg(*kop-mcherry-f-3'nanos3UTR*) transgenic line were engineered to express cytoplasmic GFP by injection of *gfp-nanos3’UTR* mRNA, [22] in addition to mCherry on their membrane. This allowed for a more reliable volume rendering by combining the cytoplasmic and membrane signals. Z-stack time lapses were obtained (13 slices x 2 μm, 25 time points in 5 sec interval). The 3D reconstruction and the volume measurement provided similar results when using connected components Plugin of the ICY software (http://icy.bioimageanalysis.org) or the Imaris 9.1.2 (Bitplane) alternative (S2 Fig). The comparison was conducted on two stacks from wild-type cells by applying a 2D median filter (half size = 3), thresholding and extracting the volume data using the “connected components function”. As the results were very similar (see S2 Fig), we used the Imaris surface function option, as it provided superior 3D representation for distinguishing blebs.

### RNA expression and bleb frequency measurements

mRNA was synthesized using the mMessage Machine kit (Ambion). RNAs were injected into the yolk of one-cell stage embryos. The experimental and control embryos belonged to the same clutch of eggs. For the data presented in S3B Fig, embryos from the Tg(*kop-mcherry-f-3'nanos3UTR*) transgenic line were injected with 100 pg of *aqp3-gfp-nos3’UTR* mRNA and imaged at 7hpf. For the bleb frequency in S3C Fig, embryos were injected with 400μM aqp1a + 400μM aqp3a morpholinos (see [20] for sequence) or 800μM of control morpholino [23]. 50pg of *gfp-nanos3’UTR* mRNA were co-injected, to verify that the embryos were indeed injected. The imaging was performed at 12–16 hpf with 25 time points acquired at 5 second intervals. Blebs were counted manually using the Fiji software.

### Analysis of the cytoplasmic flow

To conduct an analysis of the flow within the PGCs, we expressed GFP in the cytoplasm by injecting Tg(*kop-mcherry-f-3'nanos3UTR*) transgenic embryos with 100pg of *gfp-nanos3’UTR* mRNA, and then imaged the formation of blebs at a rate of one frame every two seconds. The resulting cytoplasmic streaming towards the protrusion was then analyzed using BioFlow, as described in [24]. This software extracts dense motion maps from consecutive pairs of images to highlight the redistribution of material within the cell during the evolution of a bleb.

The underlying algorithm in [24] is based on a technique known as Optical Flow (OF) but introduces several modifications. In its original version, OF makes two central assumptions to extract motion information from a pair of images. First, that the intensity of a pixel is conserved over time, i.e. a pixel in the first image can only displace to a pixel with similar intensity in the next image. Second, that the resulting flow is smooth. The purpose of this is to regularize the initially ill-posed problem. However, both assumptions are not necessarily fulfilled in biology, especially in the context of confocal microscopy. For this reason, BioFlow introduces two amendments to the seminal OF formulation. First, it considers the imaging system to take into account any possible out-of-plane flow. That is, it requires the extracted motion to fulfill a modified conservation equation for the intensity. And second, it constrains the resulting flow to behave according to a fluid-like model of the cytoplasm. The final constrained problem is then embedded into a multi-resolution scheme, yielding dense and reliable flow maps at the desired detail.

Since the expressed GFP is the source of image intensity, the algorithm acts here as a reliable flow tracker of the fluorophore molecules within the cytoplasm. In particular, no model of the whole cell or of the membrane is imposed, and the boundary velocity of the GFP signal is directly derived from the data. In addition, the proposed assumptions in Bioflow are not divergence-free and thus are able to account for both out-of-plane flow and volume loss, which is critical in the study presented here. Indeed, as presented in equations 10–12 in Boquet-Pujadas et al [24], the method is extended to cope with mass variations by relaxing the conservation equation. This is possible because divergence can also be inferred from the intensity data.

### Statistical analysis

The statistical analysis was performed using the GraphPad Prism 6 software and Microsoft Excel.

## Results and discussion

### Volume change during bleb formation

**Fig 1:**
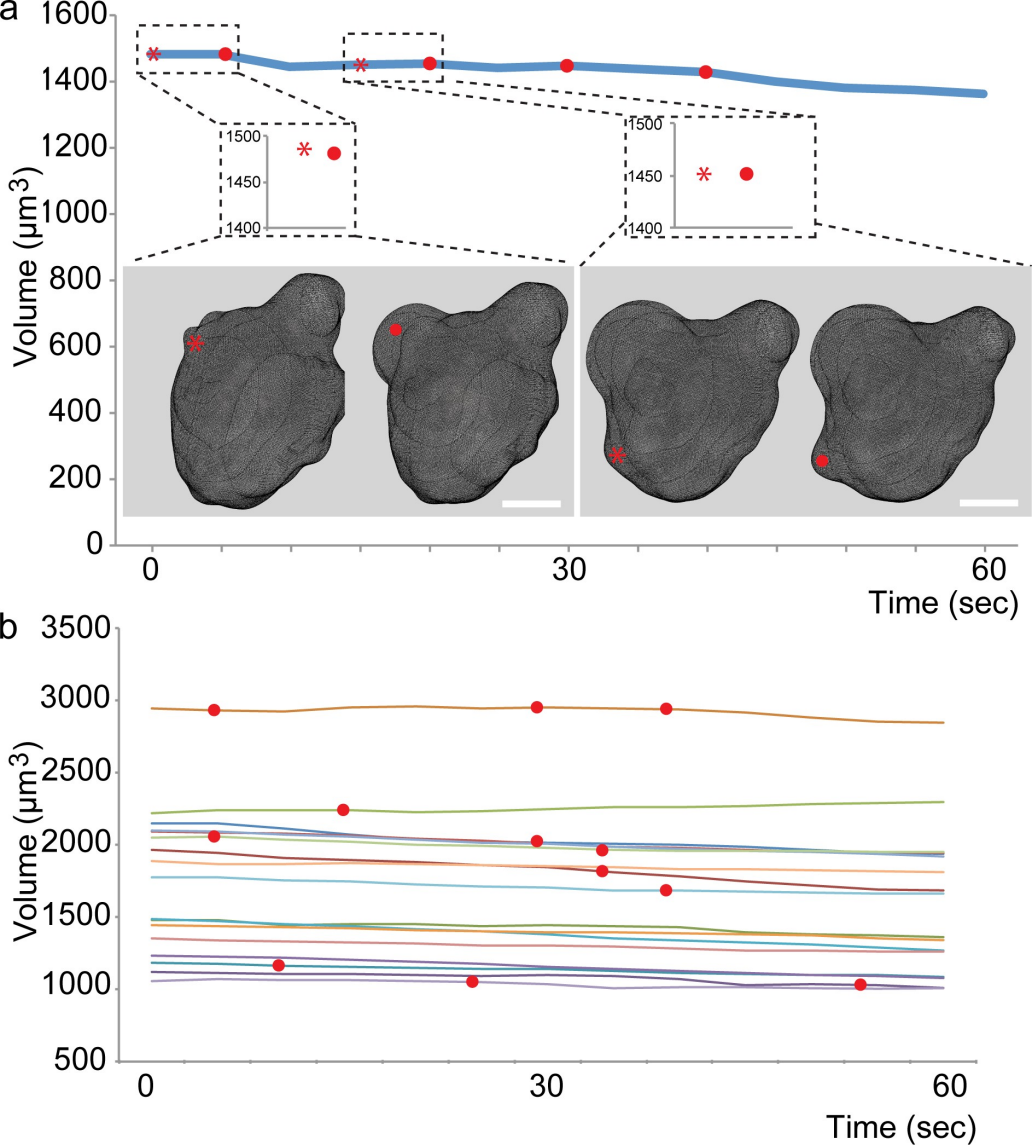
Cell volume during blebbing. (A) A graph showing the volume of a representative PGC over the course of 60 seconds. The dashed rectangles show the magnifications of pre-bleb (asterisk) and bleb (red dot) time points. The images show the corresponding 3D reconstructions. (B) The graph shows the volume of 15 control PGCs over 60 seconds, in embryos injected with 800μM of control morpholino at one cell stage. The red dots mark time points when blebs were observed. Scale bar = 5 μm.

As a first step in evaluating the possibility of volume changes in the course of cellular blebbing in PGCs, we acquired high-resolution 3-dimensional (3D) Z-stacks at a high temporal resolution (one stack per 5 seconds), employing spinning-disk confocal microscopy. 3D reconstruction of the stacks in which the PGCs were labeled with mCherry on the plasma membrane and GFP in the cytoplasm allowed us to determine the precise volume of the cells during the course of bleb formation (Fig 1A, red asterisks mark the positions where the blebs will form, and the red dots mark the expanded blebs, S1 Movie). Using this methodology, we could not detect any significant increase in cell volume linked to bleb formation events (Magnification insets in Fig 1A and 1B, 15 cells examined over 60 seconds (unlike in [17], where only pre- and post bleb values were considered). Red dots mark time points when expanded blebs were observed. We attribute the modest overall reduction in volume in some cells to a low degree of signal bleaching. These results are similar to those obtained in germ cells in which blebbing activity was inhibited by the expression of a dominant negative form of the Rho kinase (ROK) protein [11] (S1 Fig and S2 Movie). Importantly, the results obtained when employing the Imaris software were qualitatively similar to those obtained when employing the open community platform for bioimage informatics, ICY software (http://icy.bioimageanalysis.org) for analyzing the data (see examples for two cells, S3 Movie and the corresponding S2 Fig).

### The role of aquaporins in bleb formation

These results prompted us to examine a related issue. Specifically, morpholino antisense oligonucleotide-based inhibition of Aqp1a and Aqp3a protein expression in zebrafish PGCs was reported to diminish blebbing activity to the level observed in cells expressing the dominant negative form of ROK [20]. As the expression of the mRNAs encoding for Aqp1 and Aqp3 was not investigated before in zebrafish PGCs, we first examined the expression of these mRNAs in the cells using deep mRNA sequencing at the time of active PGCs migration [15]. Interestingly, at the relevant time (7 hours post fertilization) *aqp*1a RNA is not expressed in the cells of interest, while *aqp3a* mRNA is expressed at a very low level, presumably reflecting background signal (less than 1% of the PGC marker *nanos3* and less than 4% of the house-keeping gene *odc*, S3A Fig) [25]. Determining the subcellular localization of artificially expressed Aqp3a in the PGCs as performed before by Taloni *et al* [20], revealed that the fusion protein is predominantly found in what appears to be the endoplasmic reticulum (S3B Fig). Together, these observations do not provide support for the idea that the protein functions in controlling water influx into the PGCs at the relevant stages. Along the same lines, although morpholino-based phenotypes are considered less reliable without conducting a large set of control experiments and mutant analysis [26], controlled morpholino-based experiments utilizing the same morpholino species used by Taloni et al. [20] revealed no effect on bleb formation (S3C Fig and S4 Movie). Accordingly, many germ cells successfully arrived at the region of the developing gonad despite the very strong deleterious effect of the treatment on embryonic development (S3D Fig). It should be noted that even if a mild migration phenotype were detected in such a global treatment (the injected morpholino is distributed in all cells of the embryo), the effect on the somatic cells with which PGCs interact would preclude reaching conclusions regarding a specific role for Aqp1 and Aqp3 within the germ cells themselves.

### Flow of cytoplasm during bleb formation

**Fig 2:**
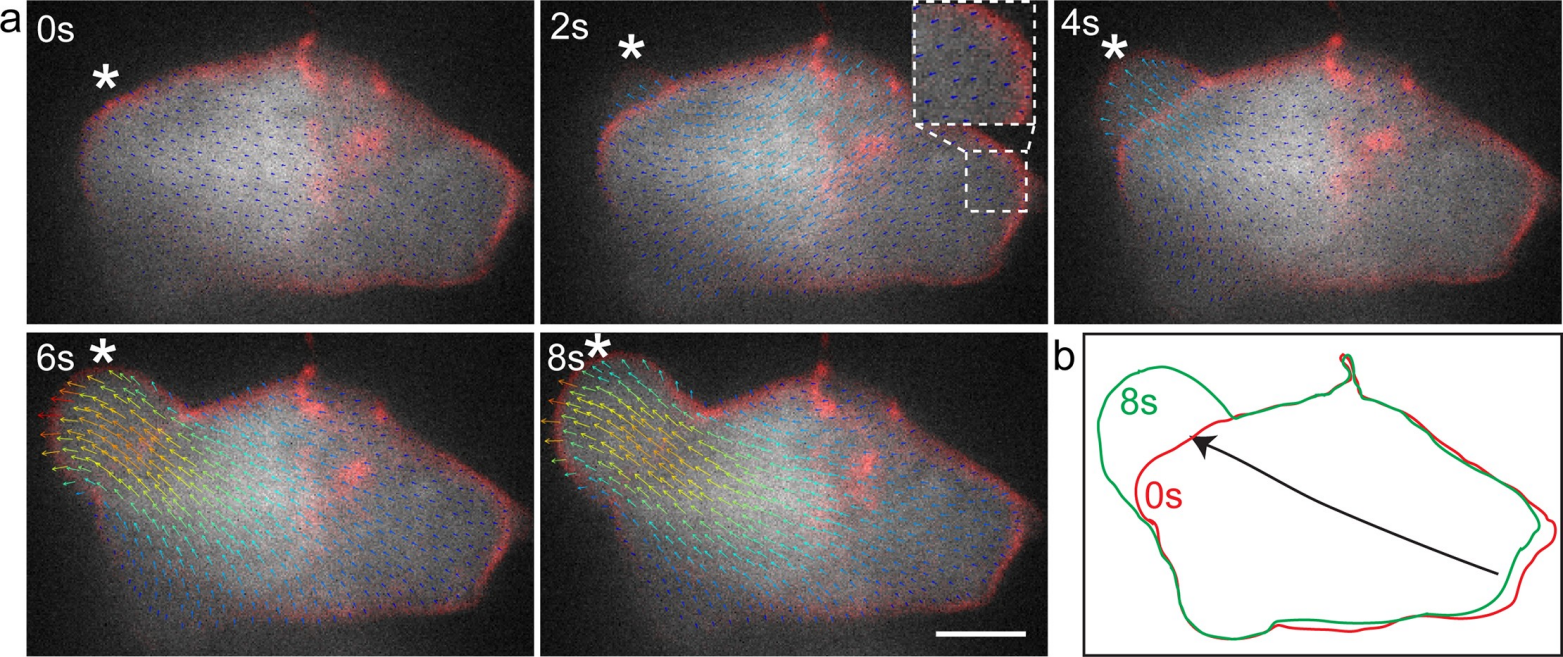
BioFlow software generated vector field analysis of cytoplasmic streaming and cell shape changes in a migrating PGC. (A) Time frames from supplementary time-lapse movie 5 showing cytoplasmic flow from the cell body into the inflating bleb. The arrows point in the direction of the flow. Higher flow speeds are depicted in red and low in blue (0.4μm/sec to 0 μm/sec respectively). Inset shows a magnification of the back of the cell, showing the flow vectors at the moment of bleb expansion. Asterisk marks the position where the bleb forms. Scale bar = 5 μm. (B) Outline of the first and last frames of panel (A) showing the contraction of the cell body concomitant with the inflation of the bleb. Arrows show the direction of the flow.

**Fig 3:**
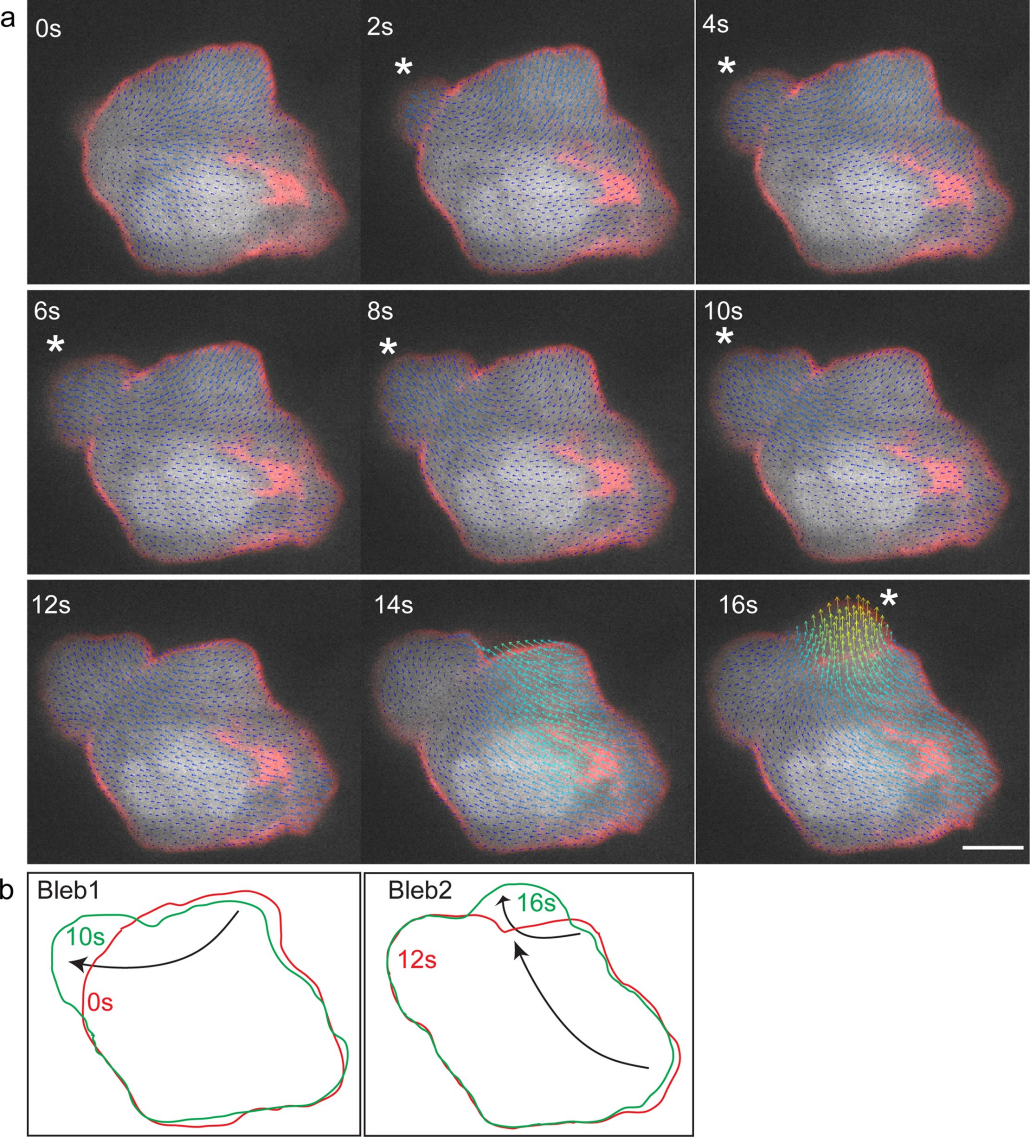
BioFlow-software-generated vector field analysis of cytoplasmic flow in a tumbling PGC. (A) Time frames from supplementary time-lapse movie 6 showing cytoplasmic flow from the cell body into the bleb for two consecutive blebs. The arrows point in the direction of the flow. Higher flow speeds are depicted in red and low in blue (0.7μm/sec to 0 μm/sec respectively). Asterisk marks the position where the first bleb (2 s) and the second bleb (16 s) form. Scale bar in A = 5 μm. (B) Outline of the first and last frames of the two blebs in panel (A) showing the contraction of the cell body co-occurring with the inflation of the bleb. Arrows show the direction of the flow.

Our findings are thus consistent with the idea that the changes in cell shape during bleb formation involve primarily internal mobilization of cytoplasm, rather than influx of water from outside the cell into the forming bleb. To examine this possibility more directly, we expressed GFP in the cytoplasm of the germ cells and followed the dynamics of the signal flow during the course of bleb formation. This analysis was conducted using the BioFlow software that allows extracting the motion of intracellular material observed using fluorescence microscopy [24]. As presented in detail in the methods section, the software extracts the optical flow (i.e. motion information) from video-microscopy data by following the images’ intensity under the constraints of fluid dynamics. In the context of this work, observing the GFP fluorescence signal level yields velocity estimates of the underlying cytoplasmic flow up to the microscope’s resolution, both in time and space. These data capture the progressive redistribution of the cytoplasm and thus allows conducting a non-invasive analysis of bleb formation. Indeed, as shown in Fig 2A and S5 Movie for a polarized migrating cell, the inflation of the bleb is accompanied by flow of cytoplasm within the cell. A clear flux of cytoplasm into the forming bleb can be observed, with the flow directed towards the periphery of the cell. Importantly, we found no indications for significant water influx at the leading edge of the cell, which would have been expected to mix or stir the cytoplasmic GFP. Interestingly, concomitant with the expansion of the bleb, retraction of the cell back can be observed, with cytoplasmic flow in the direction of the cell front (Fig 2A and 2B). We thus attribute the conservation of cell volume during bleb expansion to the concomitant retraction of other parts of the cell that compensate for the volume of cytoplasm translocated in the direction of the forming bleb. To examine this point more critically, we followed the blebbing process in cells at the “tumbling phase”, a time at which germ cells lose polarity and extend blebs in all directions [27]. Similar to the findings obtained in the case of polarized migrating cells, even when multiple blebs form, the flow of cytoplasm was directed towards the inflating protrusions, accompanied by the retraction of more distant domains in the cell (Fig 3A and 3B and S6 Movie).

## Conclusions

Our experiments reveal that the cytoplasm flow patterns are consistent with the idea that redistribution of material within the cell is responsible for bleb growth, while the volume of migrating PGCs is not significantly altered during the course of bleb formation; we could not find evidence supporting the notion that Aqp proteins are directly involved in bleb formation. Cellular blebbing is a common process utilized by migrating cells, but it is also found in a range of other processes such as apoptosis [28], generation of lumen in the vascular system [29], cytokinesis and cell spreading [30]. We consider it very likely that our findings, supporting the idea that blebs are powered by internal translocation of material, are relevant for those other contexts where blebs are found, as well as in contexts of other migration modes.

## Supporting information

S1 Fig The graph shows the volume of 18 PGCs over 60 seconds, as measured in embryos injected with mRNA encoding for DN-ROK (100 pg of *dn-rok-3'nanos3UTR*) at the one cell stage. (JPG)

S2 Fig Volume measurements using Imaris and ICY software packages. Normalized volume (V_t_/V_Avg_*100) (upper graphs) and percent volume change per time point (V_t+1_/V_t_*100–100) (lower graphs) determined for two cells (S3 Movie) using Imaris (orange lines) and ICY (blue lines). The mild decrease in the volume of the cells results from signal bleaching in the course of capturing the 3-dimensional information over time. Both algorithms revealed no volume change correlated to bleb formation.
(JPG)

S3 Fig (A) The table shows the number of reads of specific mRNAs in PGCs at 7 hours post fertilization (hpf), based on the microarray sequencing data from [25]. The data includes a PGC specific gene (*nanos3*), a housekeeping gene (*odc*) and aquaporin 1 and 3 isoforms. (B) Subcellular localization of Aqp3-GFP expressed in the PGCs employing the 3’-untranslated region of *nanos3*. (C) A graph showing the blebbing activity of PGCs in embryos injected with either 800μM of control morpholino or with 400μM Aqp1a morpholino + 400μM Aqp3a morpholino. N is the number of embryos and n represents the number of cells analyzed. The graph shows the mean and the standard deviation. (D) A low magnification image showing the morphology of 1-day old embryos treated with control and aqp 1 & 3 morpholinos. The PGCs are labeled in red. Scale bar = 5 μm.
(JPG)

S1 Movie 3D reconstruction of a PGC showing the blebbing activity. Related to Fig 1. Scale bar = 5 μm.
(AVI)

S2 Movie 3D reconstruction of a PGC expressing a dominant negative version of the ROK protein (DN-ROK). Related to S1 Fig. Scale bar = 5 μm.
(AVI)

S3 Movie 3D reconstruction of the two cells (Cell-1 and Cell-2) presented in S2 Fig, showing the blebbing activity over one minute. Scale bars = 5 μm.
(AVI)

S4 Movie 3D reconstruction of a PGC injected with morpholinos directed against the mRNAs encoding for Aqp1a and Aqp3a showing the blebbing activity. Related to S3C Fig. Scale bar = 5 μm.
(AVI)

S5 Movie Cytoplasmic flow dynamics in a migrating PGC. Vector field analysis based on cytoplasmic-GFP (Grey) in a migrating PGC. Plasma membrane is labeled in red. The arrows point in the direction of the flow. Higher flow speeds are depicted in red and low in blue. Related to Fig 2. Scale bar = 5 μm.
(AVI)

S6 Movie Cytoplasmic flow dynamics in a tumbling PGC. Vector field analysis based on cytoplasmic-GFP (Grey) with the plasma membrane labeled in red. The arrows point in the direction of the flow. Higher flow speeds are depicted in red and low in blue. Related to Fig 3. Scale bar = 5 μm.
(AVI)
