# Supplementary figures and images for: Fluid dynamics during bleb formation in migrating cells in vivo
Source: PLoS One. 2019 Feb 26;14(2):e0212699. doi: 10.1371/journal.pone.0212699 (PMC6391022; doi:10.1371/journal.pone.0212699)

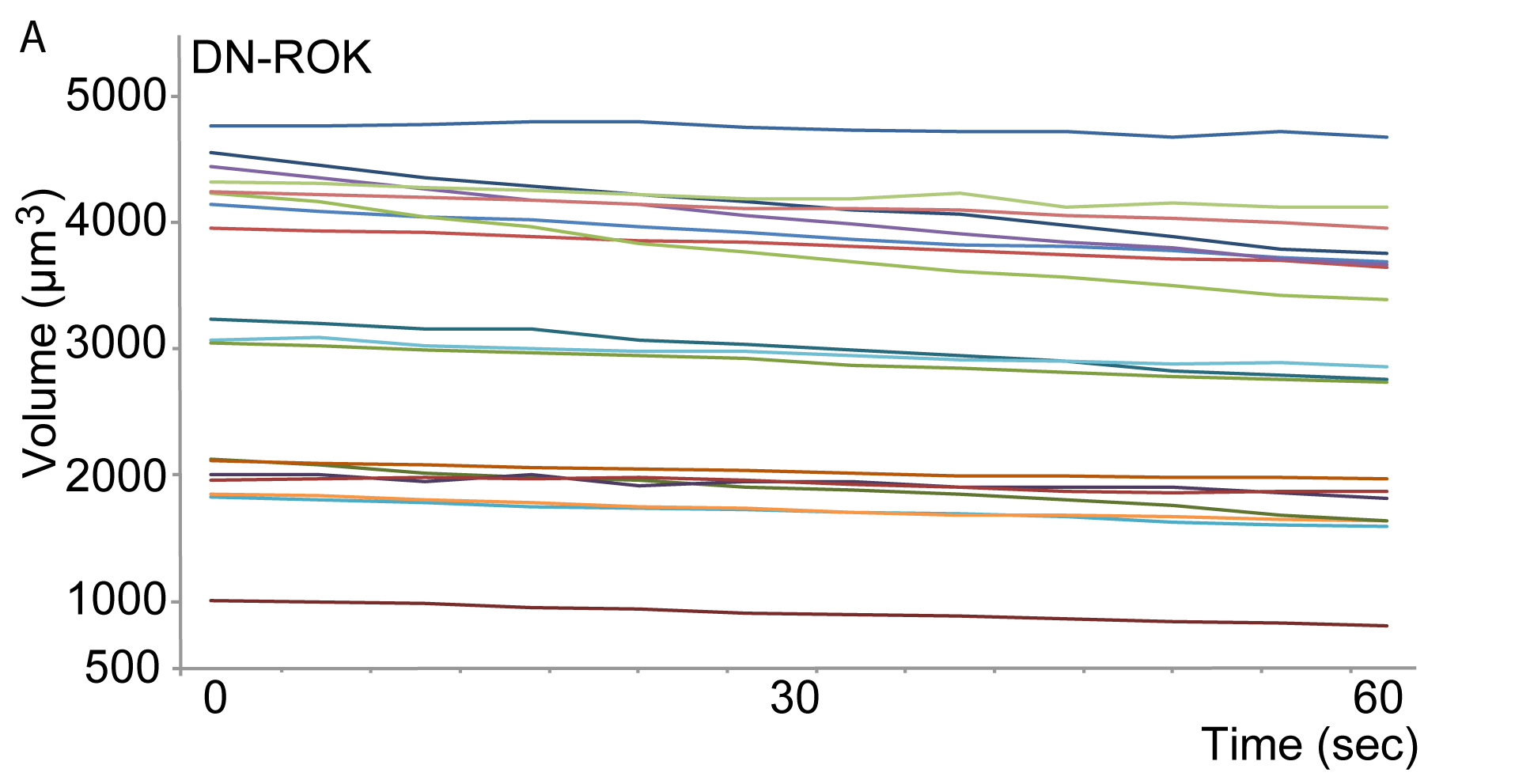

Supplement: S1 Fig — (JPG) [file pone.0212699.s001.jpg]

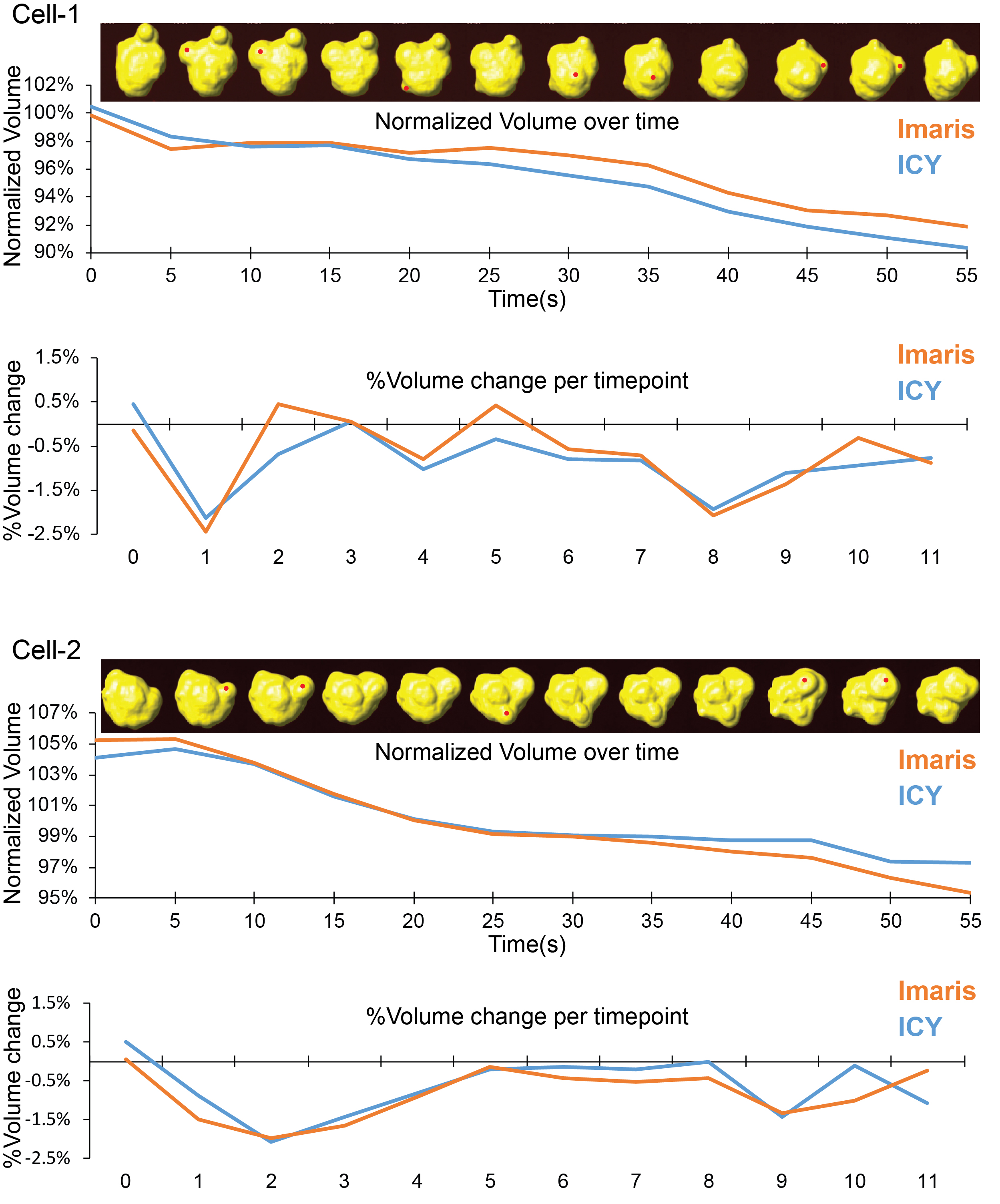

Supplement: S2 Fig — Normalized volume (Vt/VAvg*100) (upper graphs) and percent volume change per time point (Vt+1/Vt*100–100) (lower graphs) determined for two cells (S3 Movie) using Imaris (orange lines) and ICY (blue lines). The mild decrease in the volume of the cells results from signal bleaching in the course of capturing the 3-dimensional information over time. Both algorithms revealed no volume change correlated to bleb formation. (JPG) [file pone.0212699.s002.jpg]

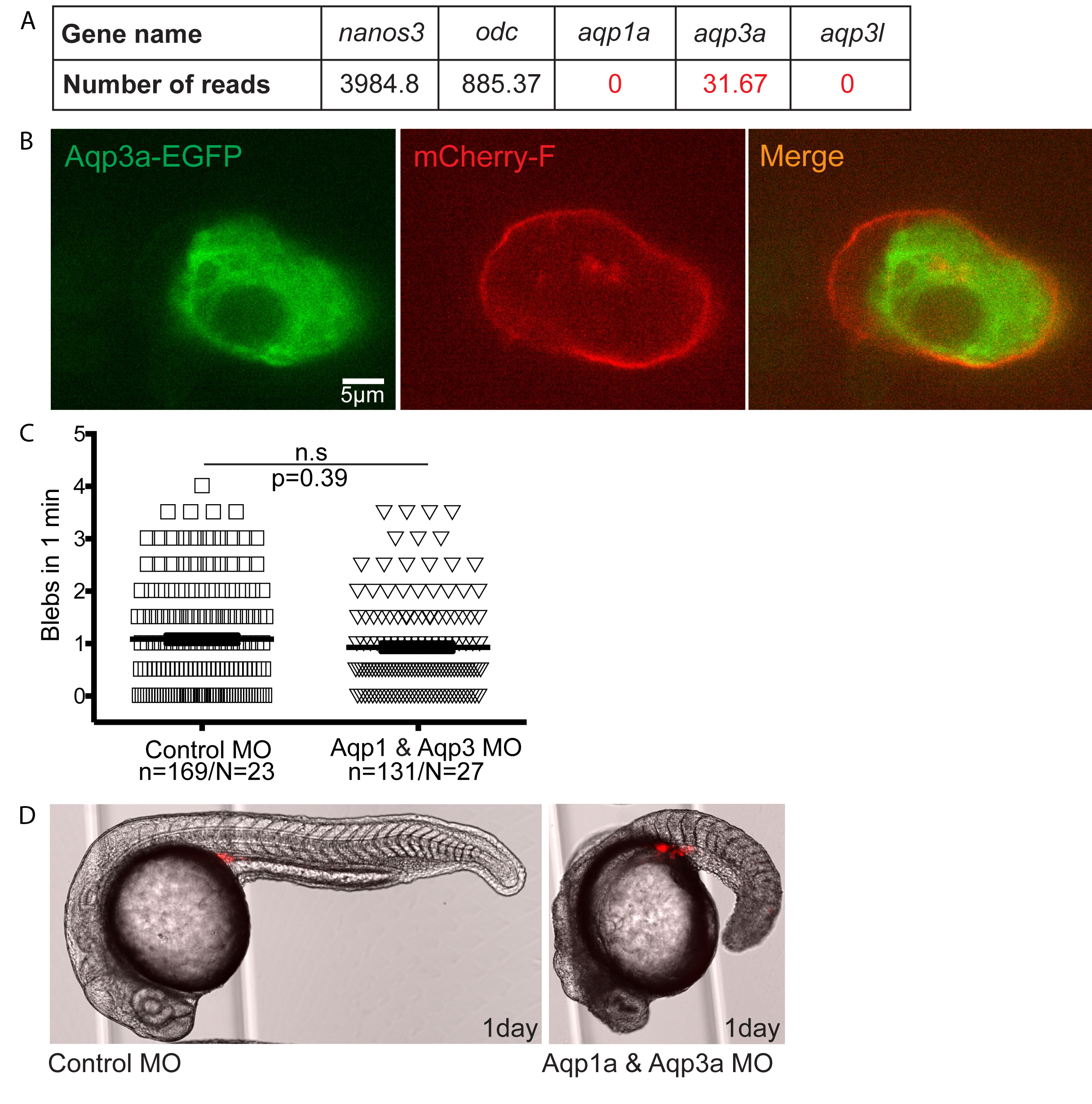

Supplement: S3 Fig — (A) The table shows the number of reads of specific mRNAs in PGCs at 7 hours post fertilization (hpf), based on the microarray sequencing data from [25]. The data includes a PGC specific gene (nanos3), a housekeeping gene (odc) and aquaporin 1 and 3 isoforms. (B) Subcellular localization of Aqp3-GFP expressed in the PGCs employing the 3’-untranslated region of nanos3. (C) A graph showing the blebbing activity of PGCs in embryos injected with either 800μM of control morpholino or with 400μM Aqp1a morpholino + 400μM Aqp3a morpholino. N is the number of embryos and n represents the number of cells analyzed. The graph shows the mean and the standard deviation. (D) A low magnification image showing the morphology of 1-day old embryos treated with control and aqp 1 & 3 morpholinos. The PGCs are labeled in red. Scale bar = 5 μm. (JPG) [file pone.0212699.s003.jpg]
